# The nature and severity of stab wounds at tertiary care hospitals in Kingdom of Saudi Arabia

**DOI:** 10.11604/pamj.2019.34.212.20533

**Published:** 2019-12-26

**Authors:** Abdulmohsen Khlaif Alenazi, Nasser Awadh Almutairi, Yousef Khalid Alhuzaimi, Saif Sulaiman Altamimi, Yasser Sulaiman Alayed, Ziad Ghanem Alanazi

**Affiliations:** 1 Prince Sattam Bin Abdulaziz University, Colleges of Medicine, Al-Kharj, Saudi Arabia

**Keywords:** Stab, wounds, wounds and injuries, Saudi Arabia

## Abstract

**Introduction:**

Fights, domestic violence and street crimes are the major causes of stab wounds in the Kingdom of Saudi Arabia (KSA). The objective of the study was to describe the nature and severity of stab wounds at a tertiary care hospital in KSA.

**Methods:**

A cross-sectional study, which included 106 patients, was conducted at the King Khalid Hospital and Prince Sultan Centre for Health Care in Al Kharj. The patients that fulfilled the inclusion criteria were recruited for the study after having confirmed their consent. The authors procured and analysed the patients’ clinical notes to obtain information that was pertinent to the study. The authors recorded all data within a Microsoft Excel document. SPSS 22.0 was utilized for statistical analysis.

**Results:**

Of 106 patients, the majority were adolescents and young adults under the age of 40 (n = 77). Eighty-seven point seven percent of patients were male and 84.0% were Saudis. Demographic details were tabulated. The top three causes were fights (20.8%) followed by domestic violence (18.9%) and street crime (17.0%). Degree of shock, stabbing zones, injury to vessels, nerves and bones, injury severity score (ISS) and Glasgow Coma Scale (GCS) were tabulated.

**Conclusion:**

The nature and severity of stab wounds should be carefully evaluated and properly managed, as these may lead to serious complications.

## Introduction

Trauma is one of the most prevalent causes of mortality worldwide, resulting in five million deaths every year [1]. Although the most common cause of death is blunt trauma caused by road traffic accidents, penetrating trauma due to gunshot or stab injuries is also a major healthcare burden in some regions of the world, e.g. the United States, Australia and South Africa [2]. In particular, stab injuries predominate in European countries, the United States and Australia [2-4]. Most often, stab wounds occur because of domestic disputes or street violence. In fact, millions of violent acts occur around the globe, but a limited number of such incidents are reported to the healthcare facilities or law enforcement [5]. A stab or puncture wound refers to a sharp force injury caused by the thrust of a sharp weapon or pointed instrument [6]. The thrusting action applies force along the long axis of the weapon, producing depth of the wound greater than its length and width. Stab wounds may result in penetrating or perforating injuries [7]. A penetrating injury refers to a puncture wound when the weapon pierces the body cavity, while a perforating wound occurs when the weapon enters from one side of the body surface and exits from the other side. Stab wounds can be suicidal, homicidal or accidental. The most common type of stab wound is reported to be homicidal followed by suicidal, while accidental stab wounds are rare [8]. The most favored weapon for a stab wound has been reported to be a kitchen knife, as this is often readily available [1]. Similarly, the most common sites of stab wounds vary in different studies. Some studies have reported chest and abdomen as the most common sites of stab wounds, while others have reported upper extremities as the most common site for stab wounds [1, 9, 10]. Penetrating stab wounds, whether homicidal or suicidal, may result in devastating or life-threatening outcomes. Such wounds can damage internal organs or vessels, especially in the chest and abdomen, leading to infection, shock and even death [2]. In one study, stab wounds accounted for 11% of deaths caused by sharp force injuries [11].

In other studies, conducted in Norway and Australia, mortality has been reported to be 2-15% [10]. Therefore, early evaluation and prompt management is of prime importance using readily available tools, such as ultrasonography (USG), computed tomography (CT), and surgical interventions [9]. Any delay in the management of patients with stab wounds may result in irrecoverable loss, especially when there is organ damage or blood loss. In the Kingdom of Saudi Arabia (KSA), trauma is a major healthcare problem. A study conducted at Jazan General Hospital (JGH) reported minor injuries as the most common type of trauma, where blunt trauma exceeded penetrating injuries. Stabbing was reported to be the most common cause of penetrating trauma [12]. Similarly, a study performed in the Arab Middle East reported mortality of 3.4% due to stab wounds [13]. A study conducted in Oman on injuries, violence and bullying among middle school students, reported 23.7% stab wounds [14]. This shows that stab wound injuries are prevalent in the Middle East and there is an urgent need to evaluate and address this issue in order to reduce morbidity, mortality, and the preventable healthcare burden. Another important aspect of stab wounds is medico-legal proceedings. An accurate history and careful examination are required to construct evidence for law enforcement. Limited studies have been conducted on stab wounds in Saudi Arabia. A few studies that have been done in the country included only a few stab injuries. As penetrating trauma is prevalent in KSA, we conducted a cross-sectional study to determine the nature and severity of stab wounds at tertiary care hospitals in KSA. Most importantly, the study also reported an injury severity score (ISS), Glasgow Coma Scale (GCS), and hypovolemic shock for stab wounds in different parts of the body. This will be a great addition to literature on stab wounds, influencing the future decisions of policy-makers regarding penetrating trauma in KSA.

## Methods

This cross-sectional study was conducted at the King Khalid Hospital and Prince Sultan Centre for Health Care in Al Kharj Saudi Arabia (KSA) in order to determine the nature and severity of stab wounds which were managed from December 2018 to March 2019. Before embarking on the research, authors conducted a comprehensive online search which was undertaken using four medical databases such as PubMed, Embase, Medline and AcademicSearch using keywords such as “stab”, “Saudi”, “KSA”, and “injury” with various Boolean operators to produce a focused and precise preliminary search result. No studies that primarily evaluated stab wounds within KSA were identified, although several studies evaluated the pattern and incidence of traumatic injuries and occupational injuries within KSA. In view of the scarcity of literature availability on the nature and severity of stab wounds within KSA, the authors set out to conduct a cross-sectional study. One hundred and six (106) patients were recruited after implementing a stringent set of inclusion and exclusion criteria. The sample size was calculated by using the Clopper-Pearson formula (i.e. at least 100 patients were required to gain a 95% confidence interval with a 5% margin of error). The inclusion criteria for this study were as follows: both male and female genders, all ages, provision of informed consent by the patient or legal guardian, residing within KSA, having presented to the King Khalid Hospital and Prince Sultan Centre for Health Care, and having at least one stab wound regardless of concomitant pathology. The exclusion criteria for this study were as follows: patients who were already admitted to an inpatient facility and patients who failed to provide legal guardian consent. The patients who were deceased either at presentation or during their in-hospital stay were also excluded from the study.

All the patients were identified at the point of presentation to the emergency department and passed through the triage, admission and discharge processes. The authors procured and analyzed the patients’ clinical notes to obtain information that was pertinent to the study such as the number, location, type and characteristics of the stab wounds, the mechanism of injury and demographic information. The authors also recorded the prognosticating factors such as the presence or absence of blood transfusions, the Glasgow Coma Scale (GCS) and the presence or absence of shock. Further details surrounding the extent of the stab injuries such as neurovascular damage, bone injury and the overall Injury Severity Score (ISS) were also collected and documented. This quantitative and qualitative data was analyzed anonymously and independently by the investigators. The researchers recorded all data within a Microsoft Excel document. SPSS 22.0 was utilized for statistical analysis, and the threshold for statistical significance was determined to be at least 0.05.

## Results

A total of 106 patients presented to the King Khalid Hospital and Prince Sultan Centre for Health Services with stab wounds between December 2018 and March 2019. The majority of these patients were adolescents and young adults under the age of 40 (n = 77). Males and females were 93 (87.7%) and 13 (12.3%), respectively. Saudi and non-Saudi participants were 89 (84.0%) and 17 (16%), respectively. These results can be referenced in Table 1 below. Regarding the prevalence of knife trauma, the top three causes were fights (20.8%) followed by domestic violence (18.9%) and street crime (17.0%). In regard to the number of stab wounds amongst the recruited patient population, most (66%) had only one stab wound. The presence or absence of first-degree shock was recorded; 49% of patients had shock, while 51% did not. These results can be referenced in Table 2 below. The distribution of the stab wounds was also recorded; the prevalence of stab wounds was highest in the regions of the head and upper limbs (21.7% each), followed by the abdomen (16.98%) and the thorax (10.38%). The prevalence of the stab wounds in other anatomical zones can be referenced in Table 3 below.

**Table 1:** Demographics

Gender		
Male	93	87.7%
Female	13	12.3%
**Nationality**		
Saudi	89	84.0%
Non-Saudi	17	16.0%
**Age category**		
Under 20	21	19.8%
20 to 30	31	29.2%
30 to 40	25	23.6%
40 to 50	12	11.3%
50 to 60	10	9.4%
Above 60	7	6.6%

**Table 2:** Types of accidents, number of wounds and shock degree

Type of accident	Gender			
	Male		Female	
Accidental	8	7.5%	0	0.0%
Domestic	20	18.9%	5	4.7%
Fight	22	20.8%	0	0.0%
Not reported	2	1.9%	0	0.0%
Psychiatric	12	11.3%	2	1.9%
Street crime	18	17.0%	1	0.9%
Substance intoxication	6	5.7%	0	0.0%
Suicidal attempt/self-harm	5	4.7%	2	1.9%
Unknown	0	0.0%	3	2.8%
**Number of stab wounds**				
1 stab	70	66.0%	10	9.4%
2 stabs	7	6.6%	1	0.9%
3 stabs	6	5.7%	0	0.0%
4 stabs	3	2.8%	0	0.0%
5 stabs	3	2.8%	2	1.9%
More than 5 stabs	4	3.8%	0	0.0%
**Shock degree**				
1^st^ degree	44	41.5%	8	7.5%
No shock	49	46.2%	5	4.7%

**Table 3:** Stabbing zones and number of stab wounds

Stabbing zone	Number of stab wounds	%
**Head**	23	21.70%
1 stab	15	14.15%
2 stabs	2	1.89%
3 stabs	2	1.89%
5 stabs	1	0.94%
6 stabs	1	0.94%
7 stabs	1	0.94%
9 stabs	1	0.94%
**Neck**	12	11.32%
1 stab	9	8.49%
2 stabs	1	0.94%
3 stabs	1	0.94%
8 stabs	1	0.94%
**Neck, Thorax**	1	0.94%
3 stabs	1	0.94%
**Neck, Abdomen, Thorax**	2	1.89%
1 stab	1	0.94%
4 stabs	1	0.94%
**Neck, Abdomen, Upper limb**	1	0.94%
5 stabs	1	0.94%
**Thorax / Back**	1	0.94%
3 stabs	1	0.94%
**Thorax**	11	10.38%
1 stab	5	4.72%
2 stabs	1	0.94%
3 stabs	1	0.94%
4 stabs	2	1.89%
5 stabs	2	1.89%
**Abdomen**	18	16.98%
1 stab	17	16.04%
2 stabs	1	0.94%
**Back**	4	3.77%
1 stab	3	2.83%
2 stabs	1	0.94%
**Upper limb**	23	21.70%
1 stab	20	18.87%
2 stabs	2	1.89%
5 stabs	1	0.94%
**Lower limb**	10	9.43%
1 stab	10	9.43%

**Figure 1:**
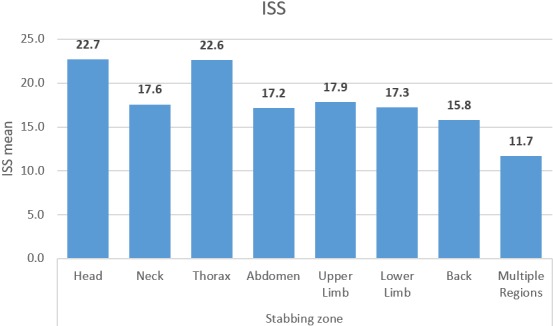
ISS means in relation to stabbing zone

**Figure 2:**
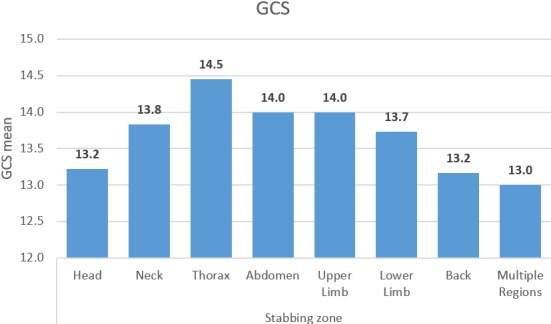
GCS means in relation to stabbing zone

The prevalence of bone and neurovascular injury was also documented by the researchers; all patients sustained vessel damage-53.8% of patients had multiple (three or more) small vessel damage. Eleven point three percent of patients had nerve damage; of these, the axillary nerve and cranial nerves had the highest prevalence (1.9% each). Eighty-one point one percent of patients did not sustain bone injury; of the remainder, clavicular and frontal bone damage was seen most often (each with a prevalence of 2.8%), followed by cranial bone damage (1.9%). The prevalence of bone and neurovascular injury for the whole cohort of patients is tabulated below in Table 4. There was a statistically significant association between the type of accident and the gender of patients (p = 0.000), and between the presence of nerve damage and gender (p = 0.047). However, these associations should be interpreted with caution, as the number of patients who fulfilled these requirements was less than five. There was no statistically significant association between the ISS and gender (p = 0.313), or between the GCS and gender (p = 0.980). Analysis of variance was performed to analyse the differences among the ISS means and GCS means in regard to the stabbing zone. This was statistically significant for the ISS (p = 0.007), but not for the GCS (p = 0.801). The means of the ISS were seen to be highest for head and thorax stab wounds, as seen in Figure 1 below. There was no statistically significant association between the means of the ISS and the type of accident (p = 0.796). However, there was a statistically significant association between the means of the ISS and the number of stab wounds (p = 0.029). The ISS means were highest for stab wounds in excess of five, as seen in Figure 1 below. The association between GCS and stabbing zone can be seen in Figure 2 below.

**Table 4:** Prevalence of bone and neurovascular injuries

	Count	Table N %
**Injured vessels**		
Small vessels	49	46.2%
Multiple small vessels	57	53.8%
**Damage of nerves**		
Yes	12	11.3%
No	94	88.7%
**Injured nerves**		
None	94	88.7%
Axillary	2	1.9%
Vagus	1	0.9%
Cervical	1	0.9%
Cranial	2	1.9%
Median	0	0.0%
Phrenic	1	0.9%
Pudendal	1	0.9%
Radial	1	0.9%
Spinal and segmental	1	0.9%
Ulnar	1	0.9%
MEDIAN	1	0.9%
**Damage of bones**		
Yes	20	18.9%
No	86	81.1%
**Injured bones**		
Ankle bone	1	0.9%
C2	1	0.9%
CARBAL #	1	0.9%
Collarbone	3	2.8%
Cranial	2	1.9%
Femur	1	0.9%
Frontal bone	3	2.8%
Frontal lobe bone fragments	1	0.9%
Hip bone	1	0.9%
Humerus	1	0.9%
Knee	1	0.9%
Nasal	1	0.9%
None	86	81.1%
Radius and Ulna	1	0.9%
Spine	1	0.9%
Ulna	1	0.9%

## Discussion

The present study describes the nature and severity of stab wounds at a tertiary care hospital in KSA. The study reveals that fights, domestic violence and street crimes are the major causes of stab wounds in Saudi Arabia. Previous studies have reported that interpersonal violence and fights are major problems all over the world, resulting in physical and psychosocial consequences [15,16]. The high prevalence of stab wounds in young people highlights the need to develop and implement certain policies to avoid such injuries. Although the frequency of stab wounds is less than other forms of trauma, such as motor vehicle crashes (MVCs) and gunshot injuries, the outcome of severe stab wounds (especially penetrating ones) may be devastating and life threatening. A retrospective study that included 6,888 patients with abdominal trauma conducted in Qatar reported frequencies of MVCs, fall from height, fall of heavy object, and penetrating wounds due to stabbing as 61%, 25%, 7% and 4.5% respectively [17]. Additionally, they reported the ISS as 17.9 ± 10. In this regard, the present study reported the ISS as 19.172 ± 6.7367 among males, and 17.154 ± 6.6564 among females. This shows that the ISS of stab wound injuries is almost similar to that of overall trauma patients. Berg *et al*. [18] studied the pattern of injuries of 617 patients with thoracoabdominal stab wounds in a longitudinal study of 16 years (1996-2011). They reported hypotension and cardiac arrest in 11% and 3.6% of patients, respectively. Similarly, they reported a GCS of <8 in 6.5% patients, and overall mortality in 3.5% patients. There is a scarcity of studies that report an overall ISS, GCS and degree of shock among patients suffering from stab wounds. However, patients with stab wounds experiencing hemorrhagic shock are at high risk of death [19].

Hokkam *et al*. [12] conducted a prospective cross-sectional study that included 1,050 patients at Jazan General Hospital (JGH) in KSA to determine different patterns of injuries presenting at the emergency department of JGH. They reported that most patients were aged between 18 and 30 years, and stab wounds were the most common cause of penetrating trauma. This shows that trauma usually occurs among adolescents and young adults, as reported in the present study. The increased probability of the penetrating nature of stab wounds makes it even more critical in terms of evaluation and management. Stab wounds may also result in major disabilities, affecting the victim’s daily routine, while increasing the healthcare burden. Al Wahbi *et al*. [20] conducted a cross sectional study that included 32 patients with vascular injuries at King Abdulaziz Medical City (KAMC), Riyadh, KSA, to determine the risk factors associated with limb loss caused by vascular injuries. They reported amputations in 46% of patients, where blunt injuries and stab/gunshot injuries accounted for 71% and 29% of injuries, respectively. Thus, stab wounds have a prominent place among critical injuries, in terms of limb loss. The strength of the present study is that it is the first study in the Middle East that covers detailed aspects of stab wounds. The weakness of the study is that it does not report the outcome of stab injuries.

## Conclusion

In conclusion, this study is of great importance, in terms of the nature and severity of stab wounds occurring in KSA, as penetrating stab wounds may damage internal organs, resulting in serious outcomes, such as loss of function, shock, major surgeries, amputation and even death. In fact, it is a great addition to the literature in terms of the nature and severity of stab wounds in KSA. Further studies are warranted to be conducted in KSA and other countries of Middle East to achieve further insights into the nature, severity and outcome of stab wounds.

### What is known about this topic

- Pattern and incidence of traumatic injuries;
- Pattern and incidence of occupational injuries;
- Evaluation and management of traumatic injuries.

### What this study adds

- The nature and severity of stab wounds in KSA;
- Associated injuries to stab wounds e.g. injury to vessels, nerves and bones;
- ISS and GCS associated with stab wound injuries.

## Competing interests

The authors declare no competing interests.
